# Atrial Flow Regulator Bridging to Heart Transplantation in Congenital Heart Disease With Severe Combined Pulmonary Hypertension

**DOI:** 10.1016/j.cjcpc.2026.05.003

**Published:** 2026-05-15

**Authors:** Mathieu Albertini, Pauline David, Hélène Nougué, Bernard Cholley, Victor Waldmann, Chekrallah Chamandi, Anne-Solene Chaussade, Leonora Du Puy-Montbrun, Guillaume Coutance, Laurence Iserin

**Affiliations:** a Centre de référence des Malformations Cardiaques Congénitales Complexes, M3C, Adult Congenital Heart Disease Unit, Centre de Recherche Cardiovasculaire de Paris, INSERM U970, Hôpital Européen Georges Pompidou, APH, Université Paris Cité, Paris, France; b Anesthesiology, Intensive Care & Postoperative Medicine, INSERM_1140, Hôpital Européen Georges Pompidou, APHP, Université Paris Cité, Paris, France

**Keywords:** atrial flow regulator, pulmonary hypertension, heart transplant, congenital heart disease

**Severe combined pulmonary hypertension is a major contraindication to isolated heart transplantation because of the high risk of post-transplant right ventricular failure. Therapeutic options are limited in patients with heart failure with preserved ejection fraction and restrictive physiology, particularly when mechanical circulatory** **support or heart-lung transplantation is not feasible. We report a patient with congenital left-sided obstructive heart disease and ineligible for transplantation due to pulmonary vascular resistance >7 Wood units. Implantation of an atrial flow regulator combined with pulmonary vasodilator therapy allowed hemodynamic improvement and ultimately successful isolated heart transplantation.**

## History of Presentation

A 48-year-old woman with complex congenital left-sided obstructive heart disease was referred for heart transplantation evaluation. She remained symptomatic (New York Heart Association class III) despite high-dose diuretics and optimal medical therapy and presented severe combined pulmonary hypertension (PH) with pulmonary vascular resistance (PVR) >7 Wood units (WU). She had been declined for isolated heart transplantation because of elevated PVR, and heart-lung transplantation was not considered given her complex surgical history, with prior thoracotomies making pleural dissection particularly challenging.

## Past Medical History

The patient was born with congenital aortic stenosis and aortic coarctation and underwent multiple prior cardiac surgeries, including neonatal repair, a Konno procedure with mechanical aortic valve replacement, and later stenting for recoarctation. She also had recurrent supraventricular arrhythmias requiring catheter ablations.

## Investigations

Transthoracic echocardiography demonstrated a nondilated left ventricle with preserved ejection fraction but restrictive filling and low cardiac output, associated with marked calcification of the mitral subvalvular apparatus. The right ventricle was hypertrophied with mildly reduced systolic function. The mean pulmonary artery pressure was 50 mm Hg.

Right heart catheterization showed right atrial pressure 9 mm Hg, pulmonary artery pressure 60/24/35 mm Hg, pulmonary artery wedge pressure 19 mm Hg, cardiac output 2.1 L/min, and PVR 7.6 WU. Dobutamine and inhaled nitric oxide testing did not initially reduce PVR below 5 WU (Table 1).

**Table 1:** Summary of the different right heart catheterizations before and after atrial flow regulator implantation (baseline) and after heart transplantation

	RHC under local anesthesia, January 2021	RHC under general anesthesia (baseline), February 2024	RHC under local anesthesia at 6 months, August 2024	RHC under local anesthesia at 9 months, November 2024	RHC at 6 months after heart transplantation, August 2025	RHC at 11 months after heart transplantation, January 2026
RA pressure (mm Hg)	12	9	14	17	15	6
RV pressure (mm Hg)	95/0/12	60/0/9	95/0/14	81/0/17	51/0/15	46/0/6
PA pressure (mm Hg)	95/38/60	60/24/35	95/31/50	81/27/44	51/23/35	46/18/31
PAWP (mm Hg)	26	19	23	25	20	17
Cardiac output (L/min)	4.5	2.1	3.6	2.8	5.15	6.65
Qp/Qs ratio	1	1	1.2	1.2	1	1
Transpulmonary gradient	34	16	27	19	15	14
Transdiastolic gradient	12	5	8	2	3	1
PVR (WU)	7.6	7.6	7.4	6.8	2.9	2.1
Treatments	Furosemide 40 mg/d, spironolactone 25 mg/d	Furosemide 250 mg/d, spironolactone 75 mg/d, dapagliflozin 10 mg/d	Furosemide 500 mg/d, spironolactone 75 mg/d, dapagliflozin 10 mg/d, sildenafil 60 mg/d Atrial flow regulator	Furosemide 750 mg/d, spironolactone 75 mg/d, dapagliflozin 10 mg/d, tadalafil 40 mg/d Atrial flow regulator	Furosemide 80 mg/d, tadalafil 40 mg/d, bosentan 250 mg/d, delexipag 1200 mg/d	Furosemide 125 mg/d, dapagliflozin 10 mg/d, tadalafil 40 mg/d, bosentan 250 mg/d

PA, pulmonary artery; PAWP, pulmonary artery wedge pressure; PVR, pulmonary vascular resistance; Qp/Qs ratio, pulmonary-to-systemic flow ratio; RA, right atrial; RHC, right heart catheterization; RV, right ventricular; WU, Wood units.

## Management

After multidisciplinary heart team discussion, a strategy aiming to unload the left atrium in order to permit pulmonary vasodilator therapy was adopted. Transseptal puncture was performed, followed by dilation of the interatrial septum using a 10-mm Armada balloon and then implantation of an 8-mm Occlutech atrial flow regulator (AFR) (Occlutech, Helsingborg, Sweden).

Pulmonary vasodilator therapy was subsequently introduced and progressively optimized together with intensive diuretic treatment. Follow-up catheterization demonstrated stable PVR but improved cardiac output (3.6 L/min). After further optimization, repeat hemodynamic assessment showed a reduction in PVR to 6.8 WU, and inhaled nitric oxide testing decreased PVR to 4.7 WU, suggesting potential transplant eligibility (Table 1).

Overall findings were consistent with severe combined PH. The elevated PVR was likely overestimated due to low cardiac output, supported by a moderate transpulmonary gradient (19 mm Hg) and a low diastolic pressure gradient (2 mm Hg) under tadalafil. Dobutamine testing was poorly tolerated, as it induced rapid atrial fibrillation with associated hemodynamic instability. Demonstration of PVR <5 WU under inhaled nitric oxide supported the feasibility of isolated heart transplantation.

A high-risk isolated heart transplantation was therefore planned despite persistent PH, with postoperative extracorporeal membrane oxygenation (ECMO) available if required. Mechanical circulatory support was not considered: left ventricular assist device implantation was precluded by severe calcification of the left ventricular papillary muscles, small ventricular cavity, restrictive physiology, and mildly impaired right ventricular function, and durable biventricular support was unsuitable given the patient’s body size.

## Outcome and Follow-up

The patient underwent heart transplantation in February 2025.

The postoperative course was complicated by septic mediastinitis and right ventricular failure requiring temporary venoarterial ECMO support. After surgical revision, targeted antibiotics, and escalation of PH therapy including triple oral therapy, right ventricular function progressively recovered and ECMO was successfully weaned.

At 6 months after transplantation, the patient reported no dyspnea and a marked improvement in quality of life compared with the pretransplant period (Kansas City Cardiomyopathy Questionnaire – 12: 37.71 vs 88.75). Right ventricular function improved, with only moderate tricuspid regurgitation requiring low-dose diuretics. PH therapy was reduced to dual therapy based on right heart catheterization findings, and this de-escalation continued at 1 year with monotherapy (Table 1).

## Discussion

The spectrum of congenital left-sided obstructive lesions may, in adulthood, evolve toward a restrictive physiology mimicking restrictive cardiomyopathy, combining heart failure with preserved ejection fraction (HFpEF) and severe combined PH. In our patient, macroscopic examination of the explanted heart revealed extensive calcification of the left ventricular papillary muscles, explaining the restrictive phenotype and progression toward pulmonary vascular disease (Fig. 1A).

**Figure 1:**
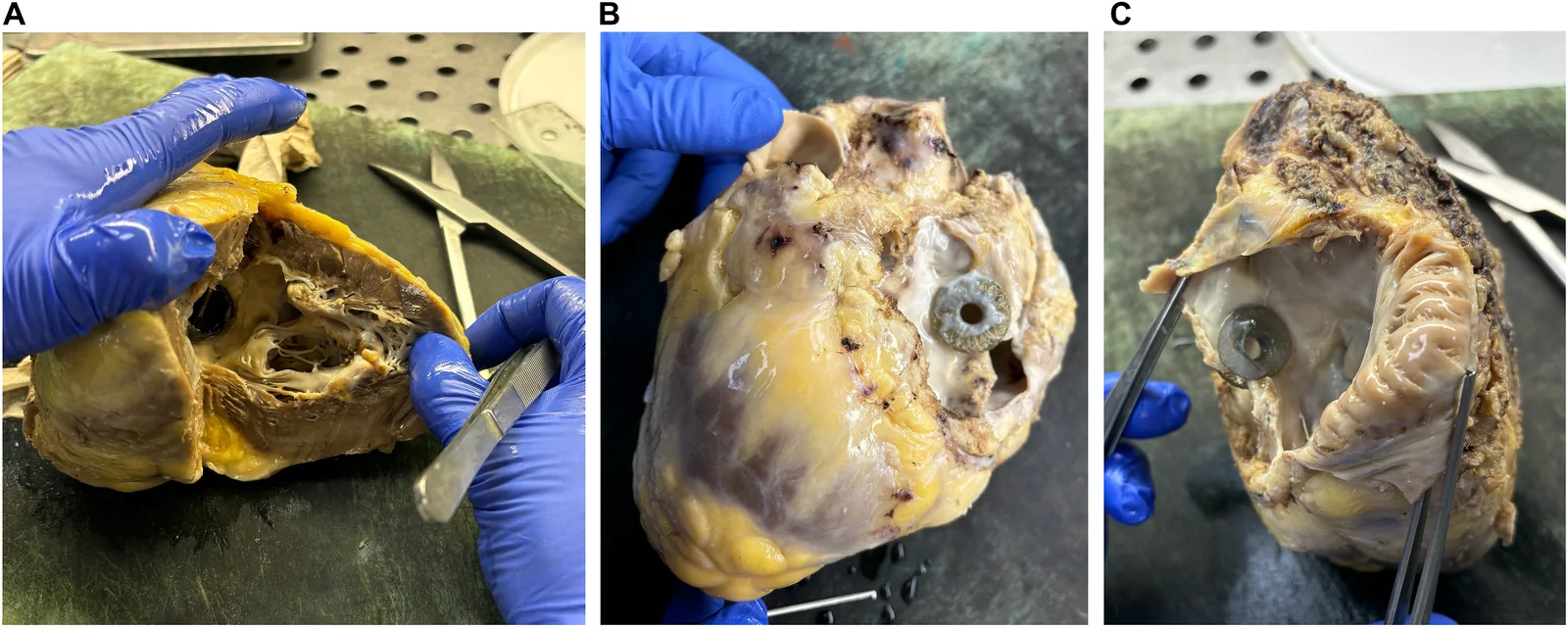
Gross examination of the explanted heart. (**A**) Macroscopic examination of the left ventricle showing extensive calcifications of the papillary muscles and the mitral subvalvular apparatus. (**B**) Visualization from the left atrium, showing no thrombus on the device and partial endothelialization. (**C**) Visualization from the right atrium, showing no thrombus on the device and partial endothelialization.

Severe combined PH remains a contraindication to isolated heart transplantation, directing these patients toward combined heart-lung transplantation. However, the limited accessibility and high operative risks associated with this type of transplant make this option relatively rare.^1^ Nonetheless, the proportion of patients with this specific profile is increasing in the population of adults with congenital heart disease but also in the context of HFpEF and restrictive cardiomyopathies. Strategies must therefore be implemented to allow a bridge to isolated heart transplantation under the best possible conditions by reducing PVR as much as possible before transplantation. Left ventricular assist device are part of these strategies, acting on the postcapillary component, but without guaranteeing a significant reduction in PVR and requiring an additional surgical procedure.^2^ Similarly, long-term biventricular assist devices may be considered, although patient size often represents a significant limitation in this context.^3^ Furthermore, after isolated heart transplantation, the risk of postoperative pulmonary hypertensive crisis is high and may require a multidisciplinary approach. Management options include PH therapies, perioperative strategies such as atrial septal defect creation with the trade-off of cyanosis, inotropic support, and, in selected cases, mechanical circulatory support such as venoarterial ECMO or pulmonary artery-left atrium devices such as the Novalung (Xenios AG, Heilbronn, Baden-Württemberg, Germany).

The Occlutech AFR is a self-expandable double-disc nitinol device with a central fenestration (Fig. 1, B and C), approved in Europe only for patients with HFpEF and heart failure with reduced ejection fraction.^4^ It may also be considered in compassionate use cases, particularly for postcapillary PH, as described in some case series, allowing functional improvement in patients.^5^ It has also been used in certain cases of precapillary PH to create a right-to-left shunt, at the cost of sustained cyanosis.

In our case, we proposed a multimodal, pharmacologic, and interventional approach, as an alternative to mechanical circulatory support strategies, for a very high-risk patient with a severely reduced cardiac output of 2.1 L/min, HFpEF, and severe combined PH with PVR >7 WU, initially deemed ineligible for isolated or combined heart transplantation due to prior surgical history.

The use of the AFR device may represent a valuable adjunctive strategy in this setting by specifically targeting the postcapillary component. By creating a controlled left-to-right shunt, the device allows decompression of the left atrium and pulmonary capillary bed, thereby reducing left-sided filling pressures and pulmonary congestion, at the expense of a moderate increase in central venous pressure. Importantly, this strategy does not induce clinically significant pulmonary hyperflow nor worsen PVR, even in the presence of maximal phosphodiesterase-5 inhibitor therapy. In patients with severe combined PH (PVR >5 WU), the coexistence of a fixed or partially reversible precapillary component may justify the use of pulmonary vasodilators. However, their introduction is often limited by the risk of exacerbating the postcapillary component through increased pulmonary blood flow and left-sided pressures, making titration challenging. In this context, left atrial unloading with AFR may provide a safer physiological framework for initiating and up-titrating pulmonary vasodilator therapy. By mitigating the rise in capillary pressure induced by vasodilators, the device allows a more effective targeting of the precapillary component while reducing the risk of left-sided decompensation. This combined approach may therefore improve treatment tolerance and facilitate optimal dosing of pulmonary vasodilators.

## Conclusions

A hybrid strategy combining AFR implantation, pulmonary vasodilator therapy, and aggressive diuretic management may allow successful bridging to isolated heart transplantation in patients with HFpEF and severe combined PH.

**Novel Teaching Points**

• Severe combined pulmonary hypertension (pulmonary vascular resistance >5 Wood units) in heart failure with preserved ejection fraction may not preclude isolated heart transplantation if partial reversibility can be achieved.
• Left atrial unloading with an atrial flow regulator may safely target the postcapillary component without inducing clinically significant pulmonary hyperflow.
• A combined pharmacologic and interventional approach may allow successful bridging to isolated heart transplantation in selected high-risk patients who are not candidates for mechanical circulatory support or heart-lung transplantation.

## Ethics Statement

The research reported has adhered to the relevant ethical guidelines.

## Patient Consent

The authors confirm that a patient consent form has been obtained for this article.

## Funding Sources

The authors have no funding sources to declare.

## Disclosures

The authors have no conflicts of interest to disclose.
